# Multiple Introductions of Rabbit Hemorrhagic Disease Virus *Lagovirus europaeus*/GI.2 in Africa

**DOI:** 10.3390/biology10090883

**Published:** 2021-09-08

**Authors:** Faten Ben Chehida, Ana M. Lopes, João V. Côrte-Real, Soufien Sghaier, Rim Aouini, Lilia Messadi, Joana Abrantes

**Affiliations:** 1 Laboratory of Microbiology, Immunology and General Pathology, Institution of Agricultural Research and Higher Education, National School of Veterinary Medicine of Sidi Thabet, University of Manouba, Sidi Thabet 2020, Tunisia; benchehida.faten@yahoo.fr (F.B.C.); rim_aouini@yahoo.fr (R.A.); lilia_messadi@yahoo.fr (L.M.); 2 CIBIO/InBIO-UP, Centro de Investigação em Biodiversidade e Recursos Genéticos, Universidade do Porto, 4485-661 Vairão, Portugal; analopes@cibio.up.pt (A.M.L.); joaocreal@cibio.up.pt (J.V.C.-R.); 3 Instituto de Ciências Biomédicas Abel Salazar (ICBAS), Unidade Multidisciplinar de Investigação Biomédica (UMIB), Universidade do Porto, 4050-313 Porto, Portugal; 4 Departamento de Biologia, Faculdade de Ciências da Universidade do Porto, 4169-007 Porto, Portugal; 5 Department of Virology, Institution of Agricultural Research and Higher Education, Tunisian Institute of Veterinary Research (IRVT), University of Tunis El Manar, Tunis 1006, Tunisia; sghaiersoufien@yahoo.fr

**Keywords:** rabbit hemorrhagic disease virus (RHDV), European rabbit (*Oryctolagus cuniculus*), GI.2, Africa

## Abstract

**Simple Summary:**

For more than 35 years, lagomorphs, which include rabbits and hares, have been severely affected by hemorrhagic disease viruses, such as the rabbit hemorrhagic disease virus (RHDV). Rabbits are important host species in the ecosystem, as they are prey of many species in the wild, are reared for meat production in several countries, and are kept as pets. Molecular characterization of RHDV has been key to detecting multiple introductions of this virus into Africa. Continued monitoring and control of the rabbit trade is assuming particular importance in containing the disease and reducing the socio-economic impact of outbreaks in Africa while rabbits are being promoted for poverty reduction programs.

**Abstract:**

Rabbit hemorrhagic disease (RHD) causes high mortality and morbidity in European rabbits (*Oryctolagus cuniculus*). In Africa, the presence of the causative agent, the rabbit hemorrhagic disease virus (RHDV), was first confirmed in 1992 (genotype *Lagovirus europaeus*/GI.1). In 2015, the new genotype *Lagovirus europaeus*/GI.2 (RHDV2/b) was detected in Tunisia. Currently, GI.2 strains are present in several North and Sub-Saharan African countries. Considerable economic losses have been observed in industrial and traditional African rabbitries due to RHDV. Like other RNA viruses, this virus presents high recombination rates, with the emergence of GI.2 being associated with a recombinant strain. Recombination events have been detected with both pathogenic (GI.1b and GII.1) and benign (GI.3 and GI.4) strains. We obtained complete genome sequences of Tunisian GI.2 strains collected between 2018 and 2020 and carried out phylogenetic analyses. The results revealed that Tunisian strains are GI.3P-GI.2 strains that were most likely introduced from Europe. In addition, the results support the occurrence of multiple introductions of GI.2 into Africa, stressing the need for characterizing complete genome sequences of the circulating lagoviruses to uncover their origin. Continued monitoring and control of rabbit trade will grant a better containment of the disease and reduce the disease-associated economic losses.

## 1. Introduction

Rabbit hemorrhagic disease virus (RHDV) is a member of the *Caliciviridae* family, genus *Lagovirus*, that causes a highly contagious and fatal disease in both domestic and wild European rabbits (*Oryctolagus cuniculus*). RHDV is a single-stranded RNA virus with a genome of ~7.4 kb organized into two open reading frames (ORF): ORF1 encodes seven non-structural proteins as well as the major structural capsid protein, VP60; and ORF2 encodes a minor structural protein, VP10 [1]. According to a recently proposed nomenclature [2], which considers a single species called “*Lagovirus europaeus*”, RHDV strains can be allocated to the *Lagovirus europaeus* genogroup I (GI) [2]. GI is further subdivided into four genotypes: GI.1 (former G1-G6) and GI.2 (RHDV2/RHDVb) regroup pathogenic strains of RHDV, while GI.3 (RCV-E1) and GI.4 (RCV-A1 and RCV-E2) comprise moderately and/or non-pathogenic strains (previously designated rabbit caliciviruses, RCVs). *Lagovirus europaeus* genogroup II corresponds to European brown hare syndrome virus pathogenic strains (GII.1) and related non-pathogenic strains (GII.2; commonly designated hare caliciviruses, HaCVs) [2].

In Africa, the appearance of GI.1 (RHDV) was first suspected in Egypt in 1988 [3]. In Tunisia, it was first detected in the south during 1989, but only confirmed in 1992 [4]. The virus spread rapidly all over the country and was therefore responsible for great economic losses in the rabbit production systems [4]. GI.2 was notified for the first time in Africa in 2015, with a first outbreak in Kairouan, Tunisia, that was followed by outbreaks in industrial rabbit farms in different governorates (governor regions) and that caused considerable economic distress [5]. In the same year, a GI.2 outbreak was reported in Benin [6] and, in 2016, multiple outbreaks were recorded in Côte d’Ivoire [6]. In 2017, Morocco reported its first outbreak [7], and Egypt in 2018 [8]. Finally, Nigeria [6,9,10], Ghana [11] and Senegal [6] had their first outbreaks in 2020.

The key role of recombination in the evolution of GI.2 is well-documented [12,13,14,15,16]. Indeed, several intergenotypic and intergenogroup recombination events between pathogenic and benign strains (GI.1b, GI.3, GI.4c, GI.4e and GII.1) have been described [12,14,16,17], including in association with the emergence of this new genotype [12]. This places GI.2 as an orphan capsid-type lagovirus. All these GI.2 recombinant strains present a recombination breakpoint at the RdRp/VP60 boundary and have the orphan GI.2 as donor for the structural proteins [12,13,14,15,16]. A second recombination breakpoint has been identified at the junction between p16 and p23, leading to the origin of triple recombinants [13].

Few studies have attempted to characterize the evolution and epidemiology of RHDV in Africa. A recent study by Rahali and co-workers showed that GI.2 is currently the main genotype circulating in Tunisia [18]; however, despite the existence of several recombinant GI.2 strains, no information was provided on their genomic make-up. Such characterization was performed for Moroccan and Nigerian strains, revealing the presence of GI.1bP-GI.2 [7] and of GI.3P-GI.2 recombinant strains [9] in those countries, respectively, indicating at least two independent introductions of GI.2 in Africa.

In this study, in order to assess the GI.2 recombinant type of the strains circulating in Tunisia and provide a more complete picture of this new genotype in Africa, we sequenced the full-length coding sequences of six strains from recent outbreaks and performed evolutionary analyses. We found that the circulating Tunisian strains are GI.3P-GI.2, but distinct from the other GI.3P-GI.2 strains that are present in Nigeria, and have a possible European origin. Furthermore, our results show that GI.2 has been introduced multiple times in Africa, highlighting the need for monitoring and characterizing the complete genome of circulating lagovirus strains.

## 2. Materials and Methods

### 2.1. Virus Samples and Genome Amplification

**Figure 1:**
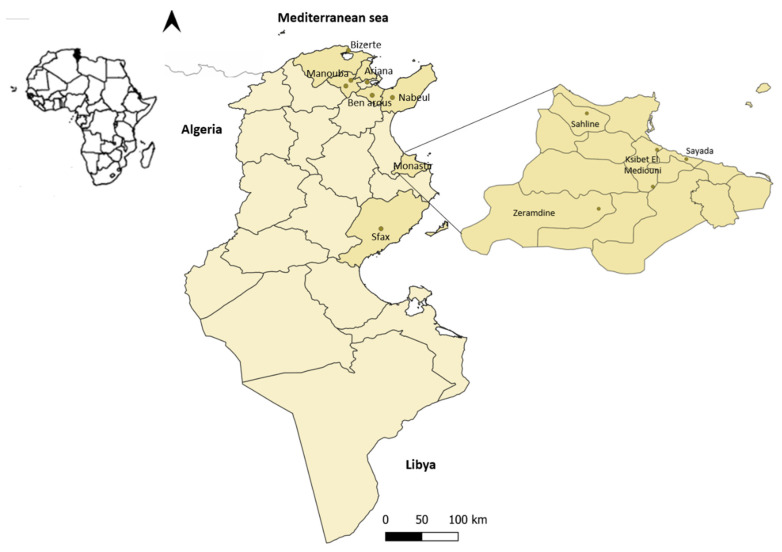
Map with the location of the RHD-suspected dead rabbits sampled from the Tunisian industrial rabbitries between 2018–2020.

Liver samples were collected from RHD-suspected dead rabbits from twelve industrial rabbitries from seven governorates of Tunisia between 2018–2020 (Figure 1, Table 1). Macroscopic lesions consisted of hemorrhages in different organs including liver, lungs and trachea; high morbidity and mortality rates were observed in these rabbitries, and the rabbits died within 24 to 48 h. RNA was extracted from 30 mg of liver homogenized in MEM medium (Dibco) using the Purelink viral RNA/DNA Mini Kit (Invitrogen), according to the provided protocol. Presence of RHDV was initially determined by RT-PCR using the Superscript One step RT-PCR System with Platinium kit (Invitrogen) with two set of primers: RHDV-F/RHDV-R, which amplifies a 348-bp portion of the VP60 gene of genotypes GI.1 and GI.2, and Fra109-F/Fra567-R, which targets a 482-bp portion of the VP60 gene of the GI.2 genotype [19].

Full coding sequences of six strains were obtained at CIBIO/InBIO, University of Porto, following a modified version of the primer-walking strategy described in [16]. Modifications included the use of additional primers and primer combinations to improve PCR amplification and sequencing (primers available from the authors upon request). RNA extraction and cDNA synthesis were performed with the RNeasy mini kit (Qiagen) and the SuperScriptTM III Reverse Transcriptase with oligo(dT) primers (Invitrogen), respectively, according to the manufacturers’ instructions. PCR products were purified and sequenced on an automatic sequencer ABI PRISM 310 Genetic Analyzer (PE Applied Biosystems) with the amplification primers. Sequences were deposited in GenBank (http://ncbi.nlm.nih.gov, accessed on 16 July 2021) under the following accession numbers: MZ913390-MZ913395.

The sequences obtained were aligned in BioEdit software version 7.0.370 [20] with all the publicly available complete coding sequences of *Lagovirus europaeus*/GI from GenBank which included genotypes GI.1-4. The final dataset consisted of 482 sequences, 7369 nucleotides in length.

### 2.2. Phylogenetic Analysis

Following the well-documented recombination hotspot in lagoviruses at the junction between the RNA-dependent RNA polymerase and the VP60 genes, the phylogenetic analysis was carried out separately for the non-structural (nucleotides 1–5295) and the structural encoding regions (nucleotides 5296–7369). Maximum-likelihood (ML) phylogenetic trees were inferred in MEGA X [21] for each partition using the best model of nucleotide substitution determined in the same software and according to the lowest AICc value (Akaike information criterion, corrected). Support for each cluster was provided from 1000 bootstrap replicates. The partial deletion (95%) option was used for handling missing data and gaps.

Genetic distances between the obtained Tunisian sequences and the remaining strains in the dataset were calculated in MEGA X. Distances were estimated separately for the non-structural and structural encoding regions using nucleotide sequences and options p-distance and partial deletion (95%) for gaps/missing data treatment.

### 2.3. Genetic Characterization and Subspecies Identification of the European Rabbits

Genetic characterization was obtained by PCR amplification and sequencing of a set of 32 single nucleotide polymorphic (SNP) markers (31 SNPs represent the nuclear genome, and one the mitochondrial genome) [22]. Statistical analyses were conducted to detect hybridization between domestic (DOM) and wild (WILD) rabbits and between *Oryctolagus cuniculus algirus* (ALG) × *Oryctolagus cuniculus cuniculus* (CUN): (i) the domestic hybridization index (HI_DOM = number of DOM alleles/number of DOM + WILD alleles) was estimated using 8 SNPs; (ii) the *O. c. algirus* and *O. c. cuniculus* hybridization index (HI_CUN = number of CUN alleles/number of CUN + ALG alleles) was estimated using 22 SNPs; (iii) a Bayesian analysis using the software Structure [23,24] to infer the genetic composition of the population and estimate the proportion of DOM, ALG and CUN genes in each individual. The analyzed rabbits were compared with a dataset available at CIBIO/InBIO with data from more than 250 individuals from 28 populations of the Iberian Peninsula as well as domestic rabbits. Subspecies identification was not possible for sample Touza 2.

## 3. Results and Discussion

In Tunisia, the first occurrence of RHDV was confirmed in 1992 and was described as an acute and highly fatal disease mainly affecting adult rabbits over 60 days of age, causing considerable economic losses in rabbit industries [4]. However, no molecular data exist from GI.1 outbreaks in this country. The novel pathogenic lagovirus GI.2, which was identified in France in 2010, rapidly reached distant places [25]. In Tunisia, GI.2 emerged in 2015, causing atypical outbreaks in domestic rabbits of all age groups, especially young rabbits, with a higher frequency of occurrence of subacute/chronic forms [19]. Molecular typing and phylogenetic analyses of the VP60 capsid gene of the circulating strains showed that from 2015 onwards GI.2 seems to be the only genotype circulating in Tunisia [18,26]. Our initial screening of the samples collected within this study are in line with this result, as all positive samples were GI.2.

Previous studies revealed that GI.2 strains are the product of several recombination events, including in those strains causing the first GI.2 outbreaks [12]. The recombination events associated with GI.2 involved pathogenic (GI.1b) and non-pathogenic strains (GI.3, GI.4c and GI.4e) as donors of the non-structural genomic region and GI.2 strains for the structural part [12,13,14,15,16]. Although not so frequent, triple recombinants have also been reported for GI.2 with an additional breakpoint at the p16/p23 boundary [13]. More recently, intergenogroup recombinants (GII.1P-GI.2) were detected in European hares (*Lepus europaeus*) [14]. Recombination greatly increases the genetic variability of the GI.2 circulating strains. In Africa, two distinct recombinants were reported: GI.1bP-GI.2 in Morocco [7] and GI.3P-GI.2 in Nigeria [9], indicating that these introductions were independent. For the remaining African countries in which GI.2 has been reported, including Tunisia, only data on the VP60 capsid gene were made available [18]. We therefore sequenced full-length coding sequences and performed recombination and phylogenetic analyses in order to fully characterize the circulating strains and attempt to pinpoint their origin.

**Figure 2:**
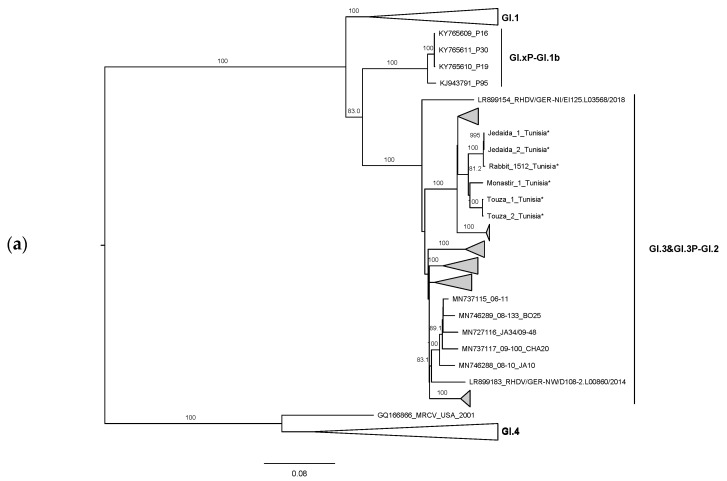

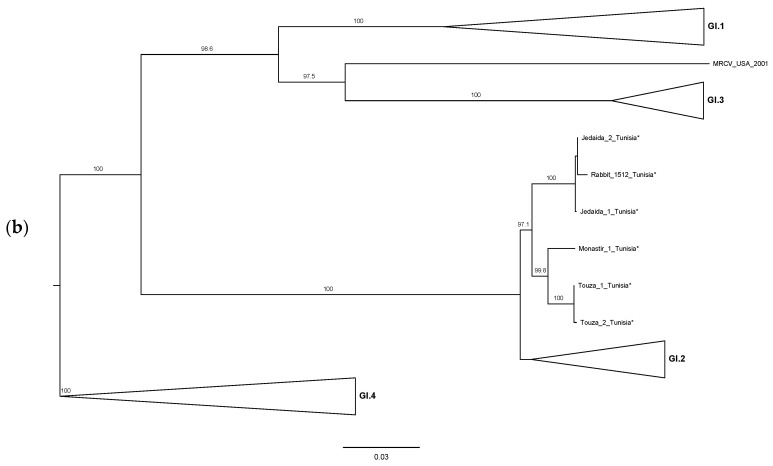
Maximum Likelihood (ML) phylogenetic trees for (**a**) the non-structural genes (*n* = 482 sequences; nucleotides 1–5295; nucleotide substitution model GTR + G + Γ4), and (**b**) the structural genes VP60 and VP10 (*n* = 482 sequences; 5296–7369; nucleotide substitution model GTR + G + Γ4). For better visualization, groups are collapsed: RHDV genotypes appear in white and GI.3P-GI.2 recombinants in grey. Horizontal branch lengths are drawn to scale of nucleotide substitutions per site and the trees are mid-point rooted. The percentage of trees in which the associated taxa clustered together was determined from 1000 bootstrap replicates and is shown next to the branches (only bootstrap values ≥70 are shown). * Sequences obtained in this study. GenBank accession numbers of the sequences used are listed in the Supplementary Materials.

The ML phylogenetic trees, constructed according to the recombination breakpoint previously identified at the RdRp/VP60 junction [12,14,15,16,17], further confirmed the recombinant origin. Indeed, for the non-structural part, the Tunisian strains grouped together in two subclusters, corresponding to strains from the Monastir governorate and strains from the Manouba governorate, within a highly supported branch containing strains belonging to GI.3 (bootstrap value 99; Figure 2a). As for the structural region, the Tunisian strains appeared again in the two closely related subclusters within the GI.2 group (bootstrap value 99; Figure 2b). Recombination was not detected at the p16/p23 junction (data not shown). The ML results confirm that Tunisian GI.2 circulating strains are GI.3P-GI.2 recombinants.

We further attempted to pinpoint the possible origin of the Tunisian strains. For this, genetic distances were calculated in MEGA X [21] for the non-structural and structural encoding regions separately using the same dataset. For both regions, the Tunisian strains presented the lowest genetic distances with strain Zar06-12 collected in 2012 in Spain (GenBank accession number KP129399): 2.69–3.97% and 2.71–2.95% for the non-structural and structural genes, respectively. The genetic distances seem to discard the hypothesis that Tunisian strains had the same origin as those from Nigeria that are also GI.3P-GI.2. Indeed, the Tunisian and Nigerian strains present 9.30–9.44% and 5.22–5.39% nucleotide differences in the non-structural and structural regions, respectively (corresponding to 2.34–2.40% and 2.07–2.44% amino acid differences in those regions; data not shown), with the Nigerian strain more closely related to other European strains (non-structural genes: 1.41% nucleotide differences with the German strain RHDV/GER-BE/EI327.L03607/2016, GenBank accession number LR899157; structural genes: 0.85% nucleotide differences with Dutch strain RHDV2-NL2016, GenBank accession number MN061492). Therefore, our results suggest independent introductions of GI.3P-GI.2 in Tunisia and Nigeria, but both with a most likely European origin. Blast analysis (blast.ncbi.nlm.nih.gov, accessed on 16 July 2021) of the capsid sequences revealed, as expected, the highest identity (~98.1–97.6%) with the Tunisian strains obtained by Rahali and co-workers [18], indicating that the strains characterized in this study do not correspond to novel introductions of GI.2 in Tunisia, but rather to the evolution of the circulating strains, and that those first Tunisian GI.2 strains were already GI.3P-GI.2.

This study shows the importance of whole genome characterization of the strains associated with outbreaks of lagoviruses occurring in new areas in order to pinpoint their origin. Indeed, and as shown, GI.2 was introduced independently in Morocco, Nigeria and Tunisia ([7,9,18], and this study), indicating the occurrence of at least three distinct introductions in Africa. Furthermore, genetic characterization and subspecies identification of the infected rabbits showed that one Tunisian rabbit was a domestic rabbit X *O. c. algirus* hybrid. This implies that, further to the illegal introduction of rabbits from Italy [18], Tunisian rabbits might have other introduction routes as natural populations of *O. c. algirus* are restricted to the southwest of the Iberian Peninsula and a few Atlantic islands [27]. The multiple introduction routes associated with rabbit trade highly increase the likelihood of the introduction of multiple GI.2 lineages.

## 4. Conclusions

In sum, our results highlight the role of (uncontrolled) animal introduction in the rapid worldwide dispersal of GI.2, and possibly also other pathogens, into naïve rabbit populations (and other existing leporids), and greatly emphasize the need for continuous surveillance and whole-genome sequencing of the lagoviruses circulating in Africa to limit the negative economic impact of GI.2 dispersal.

## Figures and Tables

**Table 1:** Origin of the rabbits sampled from the industrial rabbitries between 2018–2020.

Governorate	Region	Collection Date (Month/Year)	Sequence ID ^1^
Monastir	Sahline	10/2018	-
	Zeramdine	10/2018	Monastir 1
	Ksibet el Mediouni (north) Ksibet el Mediouni (south)	10/2018 12/2019	- Touza 1; Touza 2
	Sayada	10/2018	-
Manouba	Jedaida	12/2019	Jedaida 1; Jedaida 2
	Manouba (center)	06/2020	Rabbit 1512
Bizerte	Bizerte (south)	01/2020	-
Ben arous	Ben arous	2020	-
Ariana	Bassatine	11/2020	-
Sfax	Sfax	11/2020	-
Nabeul	Grombalia	2020	-

^1^ Samples submitted for complete genome sequencing.

## Data Availability

The data that support the findings of this study are openly available in GenBank at https://www.ncbi.nlm.nih.gov/nuccore. GenBank accession numbers are listed in the Supplementary Materials.

## Supplementary Materials

The following are available online at https://www.mdpi.com/article/10.3390/biology10090883/s1, Table S1: List of the complete genome sequences used and GenBank accession number.
