# Supplementary material for: Multiple Introductions of Rabbit Hemorrhagic Disease Virus Lagovirus europaeus/GI.2 in Africa
Source: Biology (Basel). 2021 Sep 8;10(9):883. doi: 10.3390/biology10090883 (PMC8471427; doi:10.3390/biology10090883)
Supplement: Supplementary file 1 [file biology-10-00883-s001.zip › biology-1338460-supplementary.pdf]

Table S1. List of the complete genome sequences used and GenBank accession number.

GenBank accession number	Strain name
AB300693	Hokkaido/2002/JPN_2002
AF258618	Iowa2000_USA
AF295785	Mexico89_1989
AY523410	CD/China
DQ189077	Bahrain_2001
DQ189078	Saudi_Arabia
DQ205345	JX/CHA/97_China_1997
DQ280493	ChinaWHNRH
EF363035	clonepJG-RHDV-DD06_Ramsay Island_1999
EF558572	Frankfurt12_Germany_1996
EF558573	Frankfurt5_Germany
EF558574	Wika_Germany_1996
EF558575	Ascot_UK_1992
EF558576	Jena_Germany
EF558577	Meiningen_Germany_1993
EF558578	Eisenhuttenstadt_Germany_1989
EF558579	NZ54_New Zealand_2003
EF558580	NZ61_New Zealand_2003
EF558581	Erfurt_Germany_2000
EF558582	Dachswald_Germany_2000
EF558583	Triptis_Germany_1996
EF558584	Rossi_Germany_2002
EF558585	Hagenow_Germany_1990
EF558586	Hartmannsdorf_Germany_1989
EU003578	IN-05_USA_2005
EU003579	Italy90_1990
EU003580	Korea90_1990
EU003581	NY-01_2001
EU003582	UT-01_2001
EU871528	RCV-A1_Australia_2007
GQ166866	MRCV_USA_2001
HM623309	NJ-2009_China_2009
JF412629	HYD_China_Heilongjiang_2005
JF438967	CB156_Portugal_1997
JX886001	CB194_Portugal_2006
JX886002	CB137_Portugal_1995
KF442961	Algarve1_Portugal_2013
KF442962	Algarve3_Portugal_2013
KF442963	7-13Barrancos_Portugal_2013
KF442964	10A13Barrancos_Portugal_2013
KF594473	Czechoslovakia-V351 1987
KF594474	Turretfield_06_Australia_2006
KF594475	Turretfield_07_Australia_2007
KF594476	Turretfield_09_Australia_2009
KF677011	STR2012_Poland/2012
KJ606958	Gungahlin_Australia_1998
KJ606959	Parkwood_Australia_1998
KJ943791	P95_Portugal_1996
KM115680	CBEstoi13-7_Portugal_2013
KM115681	CBEstremoz14-1_Portugal_2014

KM115682	CBEstremoz14-3_Portugal
KM115683	CBMora14-1_Portugal
KM115689	CBMontemor14-1_Portugal_2014
KM115697	CBCoruche14-1_Portugal
KM115698	CBCoruche14-2_Portugal_2014
KM115711	CBLavra10-13-1_Portugal_2013
KM115712	CBMert14-1_Portugal_2014
KM115713	CBMert14-2_Portugal_2014
KM115714	CBAlgarve14-1_Portugal
KM115715	CBAlgarve14-3_Portugal
KM115716	CBAlgarve14-4_Portugal
KM878681	N11_Spain_2011
KM979445	CBVal16_Portugal_2012
KP090974	CB110_Portugal_1994
KP090975	CB154_Portugal_1997
KP090976	CBAnd1_Spain_2012
KP129395	Rij06-12_Spain
KP129396	Seg08-12_Spain
KP129397	Tar06-12_Spain
KP129398	Zar11-11_Spain_2010
KP129399	Zar06-12_Spain
KP129400	Bar01-11_Spain_2011
KP144789	Pd_Poland/1989
KP144790	KGM_Poland/1988
KP144791	GRZ_2004_Poland_2004
KP144792	BLA_Poland/1994
KT006721	NZL/Canterbury/Lincoln/517/2013
KT006722	NZL/Canterbury/MackenzieBasin/95/2012
KT006723	NZL/HawkesBay/BoundaryStream/94/2012
KT006724	NZL/Canterbury/Hawarden/320/2013
KT006725	NZL/Otago/Queensberry/74/2013
KT006726	NZL/Otago/Taras/61/2014
KT006727	NZL/Otago/Luggate/67/2014
KT006728	NZL/Otago/Alexandra/64/2014
KT006729	NZL/Otago/Wanaka/63/2014
KT006730	NZL/Canterbury/MackenzieBasin/322/2013
KT006731	AUS/NSW/Hall/WAT1-13/2007
KT006732	AUS/NSW/Murrumbateman/BlueGums/2014
KT006733	AUS/WA/Gnowangerup/1999
KT006734	AUS/SA/FlindersRanges/1999
KT006735	AUS/WA/Bunbury/2000
KT006736	AUS/SA/Robe/2000
KT006737	AUS/SA/FlindersRanges/2002
KT006738	AUS/SA/OneTreeHill/2003
KT006739	AUS/SA/Coorong/2004
KT006740	AUS/SA/BillaKallina/2004
KT006741	AUS/SA/Woodcroft/2005
KT006742	AUS/SA/FlindersRanges/2006
KT006743	AUS/TAS/SouthArm/2007
KT006744	AUS/SA/Hillbank/2008
KT006745	AUS/SA/Bulgania/2009
KT006746	AUS/WA/WonganHills/2012
KT006747	AUS/NSW/Grafton/2013
KT280058	BlueGums-2_Australia_2015
KT280059	BLMT-2_Australia_2015

KT280060	BLMT-1_Australia_2015
KT344770	AUS/CSIRO/Master
KT344771	AUS/CSIRO/Working
KT344772	AUS/CSIRO/Release
KT344773	AUS/EMAI/Batch-1A
KT344774	AUS/EMAI/Batch-1D
KT344775	NZL/V351/Control
KU882092	GSK_Poland_1998
KU882093	MAL_Poland_1994
KU882094	OPO_Poland_2004
KU882095	ZD0_Poland_2000
KX357653	AUS/ACT/AIN-1/2009
KX357654	AUS/ACT/AIN-2/2009
KX357655	AUS/ACT/Gudg-26/2013
KX357656	AUS/ACT/Gudg-79/2014
KX357657	AUS/ACT/Gudg-89/2014
KX357658	AUS/ACT/GUN_1-240/2012
KX357659	AUS/ACT/GUN_1-29/2008
KX357660	AUS/ACT/GUN-121/2010
KX357661	AUS/ACT/GUN-159/2011
KX357662	AUS/ACT/GUN-160/2011
KX357663	AUS/ACT/GUN-238/2012
KX357664	AUS/ACT/GUN-343/2014
KX357665	AUS/ACT/GUN1-11/2007
KX357666	AUS/ACT/GUN1-22/2008
KX357667	AUS/ACT/GUN1-37/2009
KX357668	AUS/ACT/GUN1-52/2009
KX357669	AUS/ACT/GUN1-60/2010
KX357670	AUS/ACT/MtPt-2/2010
KX357671	AUS/ACT/MtPt-4/2010
KX357672	AUS/ACT/PI-1/2009
KX357673	AUS/NSW/BUR1-1/2007
KX357674	AUS/NSW/CAT_2-12/2007
KX357675	AUS/NSW/CAT_3-4/2007
KX357676	AUS/NSW/LWR-1/2010
KX357677	AUS/NSW/M2/2007
KX357678	AUS/NSW/M9/2007
KX357679	AUS/NSW/MIC3-3/2007
KX357680	AUS/NSW/MIC5-8/2007
KX357681	AUS/NSW/OAK_NT-12/2011
KX357682	AUS/NSW/OC-13/2007
KX357683	AUS/NSW/OC-15/2007
KX357684	AUS/NSW/OC-18/2007
KX357685	AUS/NSW/OC-20/2007
KX357686	AUS/NSW/OC-26/2007
KX357687	AUS/NSW/OC-33/2007
KX357688	AUS/NSW/OC-36/2007
KX357689	AUS/NSW/OC-39/2007
KX357690	AUS/NSW/OC-7/2007
KX357691	AUS/NSW/V-4/2007
KX357692	AUS/NSW/V-5/2007
KX357693	AUS/NSW/V-8/2007
KX357694	AUS/NSW/WAU-1/2009
KX357695	AUS/SA/ORA383/2008
KX357696	AUS/VIC/BEN-10/2009

KX357697	AUS/VIC/BEN-115/2010
KX357698	AUS/VIC/BEN-124/2010
KX357699	AUS/VIC/BEN-35/2009
KX357700	AUS/VIC/BEN-52/2010
KX357701	AUS/VIC/BM-2/2007
KX357702	AUS/VIC/BM-35/2009
KX357703	AUS/VIC/BM-45/2009
KX357704	AUS/VIC/BM-49/2009
KX357705	AUS/VIC/BM-60/2009
KX357706	AUS/WA/B.Hill/2013
KX357707	NZ/Southland/Gore-425A/2013
KX844830	SCH04/China_2004
KY171748	Sch07/China_2007
KY235675	QC/Canada/WIN-AH-2016-OTH-0018_2016
KY235676	MB/Canada/WIN-AH-2011-OTH-026_2011
KY235677	Mexico/2718_Mexico
KY235678	Korea/2719_Korea
KY319031	BIE_2015_Poland_2015
KY319032	GLE_2013_Poland_2013
KY319033	KRY_Poland_2004
KY319034	SKO_2013_Poland_2013
KY319035	W147/05_Poland_2005
KY437668	HB/Jingmei_China_2016
KY622127	P158_Portugal_1998
KY622128	P165_Portugal_1998
KY622129	P175_Portugal_1999
KY628306	AUS/NSW/ANN-1/2014/04
KY628307	AUS/NSW/BER-1/2013/12
KY628308	AUS/NSW/BER-10/2014/01
KY628309	AUS/NSW/BER-2/2013/12
KY628310	AUS/NSW/BER-3/2014/01
KY628311	AUS/NSW/BER-4/2014/01
KY628312	AUS/NSW/BER-8/2014/01
KY628313	AUS/NSW/BLA-1/2014/07
KY628314	AUS/NSW/GIR-1/2014/07
KY628315	AUS/NSW/GIR-2/2014/07
KY628316	AUS/NSW/KYO-1/2014/01
KY628317	AUS/NSW/OAK-1/2015/02
KY628318	AUS/NSW/OUR-1/2014/06
KY628319	AUS/NSW/OUR-2/2014/06
KY628320	AUS/NSW/WAL-1/2015/01
KY679902	L145_Poland_2004
KY679903	RED1-2013_Poland_2013
KY679904	STR2-2013_Poland_2013
KY679905	STR2014_Poland_2014
KY765609	P16_Portugal_1994
KY765610	P19_Portugal_1994
KY765611	P30_Portugal_1994/1995
LR899137	RHDV/GER-BY/EI53-2.L03573/2019
LR899138	RHDV/GER-SN/EI67.L03566/2017
LR899139	RHDV/GER-BE/EI117-2.L03571/2018
LR899141	RHDV/GER-NI/EI129-10.L03595/2019 (European hare)
LR899142***	RHDV/GER-NW/EI17-1.L03577/2019 (European hare)
LR899143	RHDV/GER-HE/EI126-2.L03569/2018
LR899144	RHDV/GER-BY/EI06-1.L03565/2019

LR899145	RHDV/GER-BB/EI16-2.L03579/2019
LR899146	RHDV/GER-BE/EI83-2.L03575/2019
LR899147	RHDV/GER-BB/EI44.L03567/201
LR899148	RHDV/GER-BE/EI48.L03572/2019
LR899149	RHDV/GER-BE/EI175-1.L03570/2017
LR899150	RHDV/GER-RP/EI73-7.L03578/2018
LR899151	RHDV/GER-BE/EI31.L03574/2019
LR899153	RHDV/GER-NW/EI121.L03597/2017
LR899154	RHDV/GER-NI/EI125.L03568/2018
LR899155	RHDV/GER-RP/EI73-1.L03576/2018
LR899156	RHDV/GER-NI/EI07.L03593/2020
LR899157	RHDV/GER-BE/EI327.L03607/2016
LR899158	RHDV/GER-TH/EI15-1.L03598/2015
LR899159	RHDV/GER-NW/EI106.L03602/2016
LR899160	RHDV/GER-SN/EI176-1.L03604/2016
LR899161	RHDV/GER-NW/D102-2.L00436/2013
LR899162	RHDV/GER-BB/D66-15.L03609/2015
LR899163	RHDV/GER-NW/EI70-1.L03601/2016
LR899164	RHDV/GER-BW/EI11-5.L03608/2018
LR899165	RHDV/GER-TH/EI145-12.L03603/2016
LR899166	RHDV/GER-MV/EI220.L03605/2016
LR899167	RHDV/GER-NW/D160-1.L03612/2015
LR899168	RHDV/GER-SN/D166.L03611/2015
LR899169	RHDV/GER-SN/EI297-1.L03606/2016
LR899170	RHDV/GER-NW/EI15-7.L03600/2016
LR899172	RHDV/GER-SN/D107.L00439/2013
LR899173	RHDV/GER-RP/D87-2.L03610/2015
LR899174	RHDV/GER-TH/EI15-3.L03599/2016
LR899175	RHDV/GER-RP/D24.L00713/2014
LR899176	RHDV/GER-NW/D102-1.L00435/2013
LR899177	RHDV/GER-SN/D105-2.L00438/2013
LR899179	RHDV/GER-SH/D51-2.L00912/2014
LR899180	RHDV/GER-NW/D123-3.L00914/2014
LR899181	RHDV/GER-NW/D46-2.L00718/2014
LR899183	RHDV/GER-NW/D108-2.L00860/2014
LR899184	RHDV/GER-NW/D44.L00716/2014
LR899186	RHDV/GER-NW/D61-2.L00910/2014
LR899189	RHDV/GER-NW/D51-1.L00911/2014
LR899190	RHDV/GER-SN/EI205.L03564/2017
LR899191	RHDV/GER-TH/D101-2.L00903/2014
LR899192	RHDV/GER-BY/D35.L00714/2014
LT168844	13-165_France_2013
M67473	FRG_Germany_1989
MF407651	CBPico17-1_Portugal_2017
MF407652	CBPico17-2_Portugal_2017
MF407653	16PLM1_Spain_2016
MF407654	PSM2_Portugal_2016
MF407655	CBMad17-1_Portugal_2017
MF407656	CBMad17-2_Portugal_2017
MF407657	CBMad17-3_Portugal_2017
MF421563	AUS/ACT/BLMT-3/2015
MF421564	AUS/ACT/AIN-3/2015
MF421565	AUS/TAS/BRK-1/2016
MF421566	AUS/VIC/CWS-1/2016

MF421567	AUS/WA/GER-1/2016
MF421568	AUS/WA/GER-2/2016
MF421569	AUS/TAS/GRA-1/2016
MF421570	AUS/TAS/GEE-2/2016
MF421571	AUS/WA/GER-3/2016
MF421572	AUS/WA/GER-4/2016
MF421573	AUS/WA/GER-5/2016
MF421574	AUS/SA/MEA-1/2016
MF421575	AUS/SA/MUN-2/2016
MF421576	AUS/SA/TIN-2/2016
MF421577	AUS/SA/WIT-1/2016
MF421578	AUS/VIC/HAM-4/2016
MF421579	AUS/ACT/ATH-1/2015
MF421580	AUS/ACT/ARA-1/2015
MF421581	AUS/ACT/TUG-1/2015
MF421582	AUS/ACT/TUG-3/2015
MF421583	AUS/ACT/HACK-1/2015
MF421584	AUS/ACT/BLMT-6/2015
MF421585	AUS/ACT/MFBM-2/2015
MF421586	AUS/ACT/WC-2/2016
MF421587	AUS/ACT/MAJ-1/2016
MF421588	AUS/NSW/BIB-1/2016
MF421589	AUS/NSW/RUS-1/2016
MF421590	AUS/NSW/KYE-1/2016
MF421591	AUS/NSW/KYE-2/2016
MF421592	AUS/NSW/BG-5/2016
MF421593	AUS/NSW/BRH-1/2016
MF421594	AUS/NSW/BG-6/2016
MF421595	AUS/NSW/SGA-1/2016
MF421596	AUS/NSW/BG-7/2016
MF421597	AUS/SA/PAR-1/2015
MF421598	AUS/SA/CRA-1/2016
MF421599	AUS/SA/LYN-1/2016
MF421600	AUS/SA/MUN-1/2016
MF421601	AUS/SA/COO-1/2016
MF421602	AUS/SA/MAC-1/2016
MF421603	AUS/SA/KAL-1/2016
MF421604	AUS/SA/KAL-2/2016
MF421605	AUS/SA/LH-1/2016
MF421606	AUS/SA/MAC-2/2016
MF421607	AUS/SA/CV-1/2016
MF421608	AUS/SA/TUR-1607/2016
MF421609	AUS/SA/TUR-1606/2016
MF421610	AUS/SA/STR-1/2016
MF421611	AUS/SA/KAL-3/2016
MF421612	AUS/SA/MTO-2/2016
MF421613	AUS/SA/BLAK-1/2016
MF421614	AUS/SA/WS-1/2016
MF421615	AUS/SA/MTO-1/2016
MF421616	AUS/SA/LEA-1/2016
MF421617	AUS/SA/CRK-1/2016
MF421618	AUS/SA/BRA-1/2016

MF421619	AUS/SA/COL-1/2016
MF421620	AUS/SA/COL-2/2016
MF421621	AUS/SA/ORO-1/2016
MF421622	AUS/VIC/HAM-1/2016
MF421623	AUS/VIC/HAM-2/2016
MF421624	AUS/VIC/TAW-1/2016
MF421625	AUS/VIC/TAW-2/2016
MF421626	AUS/VIC/HAM-3/2016
MF421627	AUS/VIC/BAH-1/2016
MF421628	AUS/WA/PIC-1/2016
MF421629	AUS/WA/CHI-1/2016
MF421630	AUS/WA/CHI-2/2016
MF421631	AUS/WA/PIC-2/2016
MF421632	AUS/WA/WNN-1/2016
MF421633	AUS/WA/MTBA-1/2016
MF421634	AUS/WA/MTBA-2/2016
MF421635	AUS/WA/WNP-1/2016
MF421636	AUS/ACT/HAR-1/2015
MF421637	AUS/ACT/THE-1/2016
MF421638	AUS/ACT/BON-2/2016
MF421639	AUS/NSW/TAR-2/2015
MF421640	AUS/NSW/MER-1/2015
MF421641	AUS/NSW/BOC-1/2015
MF421642	AUS/NSW/COW-1/2015
MF421643	AUS/NSW/ORAN-1/2015
MF421644	AUS/NSW/WES-1/2015
MF421645	AUS/NSW/EOR-1/2015
MF421646	AUS/NSW/HIL-1/2015
MF421647	AUS/NSW/BRO-1/2015
MF421648	AUS/NSW/BRO-2/2015
MF421649	AUS/NSW/BEGA-1/2015
MF421650	AUS/NSW/BEGA-2/2015
MF421651	AUS/NSW/FC-1/2015
MF421652	AUS/NSW/GOU-1/2015
MF421653	AUS/NSW/BERK-1/2016
MF421654	AUS/NSW/SWI-1/2016
MF421655	AUS/NSW/COB-1/2016
MF421656	AUS/NSW/MOL-1/2016
MF421657	AUS/NSW/KUL-1/2016
MF421658	AUS/NSW/CAR-4/2016
MF421659	AUS/NT/DAR-1/2015
MF421660	AUS/NT/ASP-1/2016
MF421661	AUS/SA/MBR-1/2015
MF421662	AUS/SA/ADL-1/2016
MF421663	AUS/SA/NUR-1/2016
MF421664	AUS/SA/ONK-1/2016
MF421665	AUS/SA/MUN-3/2016
MF421666	AUS/SA/MIL-1/2016
MF421667	AUS/SA/NAI-1/2016
MF421668	AUS/SA/ROB-1/2016
MF421669	AUS/SA/REY-1/2016
MF421670	AUS/SA/REY-2/2016

MF421671	AUS/SA/KOO-1/2016
MF421672	AUS/SA/GLE-1/2016
MF421673	AUS/SA/BAL-2/2016
MF421674	AUS/SA/BAL-3/2016
MF421675	AUS/TAS/SPR-1/2016
MF421676	AUS/VIC/KERANG-1/2015
MF421677	AUS/VIC/WAN-1/2015
MF421678	AUS/VIC/WIL-1/2015
MF421679	AUS/VIC/WIL-2/2015
MF421680	AUS/VIC/NEW-1/2016
MF421681	AUS/VIC/NEW-3/2016
MF421682	AUS/VIC/NEW-2/2016
MF421683	AUS/VIC/WAR-2/2016
MF421684	AUS/VIC/AC-1/2016
MF421685	AUS/VIC/AC-2/2016
MF421686	AUS/VIC/DUN-1/2016
MF421687	AUS/VIC/DUN-2/2016
MF421688	AUS/VIC/DUN-3/2016
MF421689	AUS/VIC/AWA-4/2016
MF421690	AUS/VIC/THO-3/2016
MF421691	AUS/VIC/AWA-1/2016
MF421692	AUS/VIC/STO-1/2016
MF421693	AUS/VIC/MLB-1/2016
MF421694	AUS/VIC/CLC-1/2016
MF421695	AUS/VIC/DON-1/2016
MF421696	AUS/VIC/CAM-2/2016
MF421697	AUS/WA/YOR-1/2016
MF421698	AUS/WA/PTH-3/2016
MF421699	AUS/WA/ALB-3/2016
MF421700	AUS/WA/LES-1/2016
MF421701	AUS/NSW/YAR-1/2015
MF598301	K5_08Q712_BatchRelease1/2008
MF598302	AUS/NSW/CAR-3/2016
MG602005	BBI_Poland_Mazowieckie_Apr-2017
MG602006	RED2016_Poland_Lodzkie_Set-2016
MG602007	VMS_Poland_West Pomeranian_Jun-2017
MG763936	SOS089_Portugal_2014
MG763937	SOS125_Portugal_2014
MG763938	SOS129_Portugal_2014
MG763939	SOS133_Portugal_2014
MG763940	SOS137_Portugal_2015
MG763941	SOS140_Portugal_2015
MG763942	SOS148_Portugal_2015
MG763943	SOS149_Portugal_2015
MG763944	SOS150_Portugal_2015
MG763945	SOS151_Portugal_2015
MG763946	SOS155_Portugal_2015
MG763947	SOS158_Portugal_2015
MG763948	SOS164_Portugal_2015
MG763949	SOS173_Portugal_2015
MG763950	SOS404_Portugal_2015
MG763951	SOS468_Portugal_2015
MG763952	SOS473_Portugal_2015

MG763953	SOS474_Portugal_2015
MG763954	SOS492_Portugal_2016
MH159170	A17-62_Morocco_2017
MH159171	A17-69_Morocco_2017
MH159172	A17-63_Morocco_2017
MH159173	A17-73_Morocco_2017
MH190418	00-21_G5
MK521927	Buckbys Road/Tasmania/Sarcophilus_harrisii/2017/DN120446
MK895974	China_2014
MN061492	NL2016_Netherlands
MN478485	Alpine_musk_deer_China_2011
MN737113	10-28_France_2010
MN737114	10-32_France_2010
MN737115	06-11_France_2006
MN737116	JA34/09-48_France_2009
MN737117	CHA20/09-100_France_2009
MN738377	16-35_France_2016
MN746288	JA10/08-10_France_2008
MN746289	BO25/08-133_France_2008
MN786321	16-36_France_2016
MN853658	F77-3_Poland_2015
MN853659	LIB_Poland_2018
MN853660	PIN_Poland_2018
MN853661	WAK_Poland_2018
MN901451	Bremerhaven-17_Lepus_timidus_Germany_2017
MT506233	RHDV2/Apr2020/TX1_USA_2020
MT506234	RHDV2/Mar2020/NM1_USA_2020
MT506235	RHDV2/Mar2020/NY2_USA_2020
MT506236	RHDV2/Mar2020/NY1_USA_2020
MT506237	RHDV2/Apr2020/AZ1_USA_2020
MT586027	SC2020/0401_China_2020
MT628287	95-10_France_1995
MT628288	05-01_G5_France_2005
MT628289	09-02_France_2009
MT628290	09-03_G5R_France_2009
MT628291	16-09_France_2016
MT819374	96-VLT000113_Sweden_1996
MT819375	03-VLT001218_Sweden_2003
MT819376	06-VLT001843_Sweden_2006
MT819377	12-VLT000099_Sweden_2012
MT819378	12-VLT000101_Sweden_2012
MT819379	10-VLT001467_Sweden_2010
MT833874	Vulpes_vulpes_feces/Australia_2019
MW123059	RT5-1_Nigeria_August_2020
MW194928	GI.2/SG-NParks/2020/M54-9_Singapore_2020
MW467791	AUS/GI.2_master_2016
U54983	RHDV-V351_1987
X87607	BS89_Italy_1989
Z29514	SD_France_1989_
Z49271	RHDV-AST89_1989
MZ913390	Jedaida_1/TUN/2019
MZ913391	Jedaida_2/TUN/2019
MZ913392	Monastir_1/TUN/2018
MZ913394	Touza_1/TUN/2019
MZ913395	Touza_2/TUN/2019

MZ913393	Rabbit_1512/TUN/2020
